# *Eurosurveillance* reviewers in 2020

**DOI:** 10.2807/1560-7917.ES.2021.26.1.2101071

**Published:** 2021-01-07

**Authors:** Eurosurveillance editorial team

**Affiliations:** 1 European Centre for Disease Prevention and Control (ECDC), Stockholm, Sweden

**Keywords:** peer-review

The editorial team is grateful to all the experts who dedicated their time to peer-review our articles over the past 12 months. We wish to thank them and all those whose names are not listed below but gave helpful advice and support. We apologise to any reviewers whose names we may have missed.

Our reviewers in 2020 were:

Mohamed Abbas; Stephan Aberle; Muna Abu Sin; Mary Adams; Karam Adel Ali; Cornelia Adlhoch; Karoline Aebi-Popp; Seven Johannes Sam Aghdassi; Antonella Agodi; Lis Alban; Jan Albert; Sheikh Taslim Ali; Franz Allerberger; Erik Alm; Christian Althaus; Matthias an der Heiden; Maria an der Heiden; Claire Anderson; Brito Anderson; Raiees Andrabi; Nick Andrews; Xanthi Andrianou; Denise Antona; Alberto Antonelli; Habibollah Arefian; Wouter Arrazola de Oñate; Tommi Asikainen; Olle Aspevall; Barry Atkinson; Erik Aurell; Sabrina Bacci; Agoritsa Baka; Craig Baker-Austin; Tamas Bakonyi; Sooria Balasegaram; Koye Balogun; Miguel Bao-Dominguez; Osamah Barasheed; Cyril Barbezange; Ian Barr; Luisa Barzon; Denis Bauer; Gabrielle Beaudry; Julien Beauté; Cecile Beck; Philippe Belanger; Edward Belongia; Justus Benzler; Anne Berger-Carbonne; Isha Berry; Marianne Besnard; Philippe Beutels; Stuart Blacksell; Hubert Blain; Evan M. Bloch; Seth Blumberg; Nicki Boddington; Pierre-Yves Boelle; Magnus Boman; Idesbald Boone; Martin Bootsma; David Boulware; Nicola Luigi Bragazzi; Arne Brantsæter; Eeva Broberg; Stefan Brockmann; Tal Brosh-Nissimov; Lisa Brouwers; Kevin Brown; Mathias Bruyand; Robin Bruyndonckx; Mia Brytting; Udo Buchholz; Niccolò Buetti; Nicholas Bundle; Thierry Burnouf; Karen Burns; Valeria Cagno; Worth Calfee; Alessandra Carattoli; Yehuda Carmeli; AnnaSara Carnahan; Gail Carson; Jordi Casabona; Geraldine Casey; Jesus Castilla Catalan; Giancarlo Ceccarelli; André Charlett; Remi Charrel; Yee-Chun Chen; Hua Chen; Nikoloz Chkhartishvili; Eun Hwa Choi; Anissa Chouikha; Gerardo Chowell; Daniel Chu; Bruno Ciancio; Sandra Ciesek; Milan Cizman; Eric C. J. Claas; Hannah Clapham; Luigi Codecasa; Rosalind Lucy Coleman; Vittoria Colizza; Alex Cook; Teresa Coque; Samuel Cordey; Victor Corman; Laura Cornelissen; Benjamin Cowling; Natasha Crowcroft; Fortunato D'Ancona; Gavin Dabrera; Niklas Danielsson; François Danion; Costas Danis; Masoud Dara; Siddhartha Sankar Datta; Helena de Carvalho Gomes; Birgitta de Jong; Ana Maria de Roda Husman; Gaston De Serres; Gerard de Vries; Genevieve Deceuninck; Valerie Delpech; David Denning; Sara Dequeker; Jean-Claude Desenclos; Simona Di Giambenedetto; Sabine Diedrich; Michaela Diercke; Joakim Dillner; W Dimech; Gerhard Dobler; Roger Dodd; Jennifer Dowd; Timothee Dub; Sandra Dudareva-Vizule; Dominic Dwyer; Isabella Eckerle; Tim Eckmanns; Michael Edelstein; Paul Effler; Maren Eggers; Martin Eichner; Jorge Eiras; David Elliman; Mallory Ellingson; Andrea Endimiani; Theresa Enkirch; Daniel Faensen; Darryl Falzarano; Ugo Fedeli; John Ferguson; Virginie Ferre; Siri Laura Feruglio; Julie Figoni; Antonietta Filia; Bastian Fischer; David Fisman; Brendan Flannery; Per Follin; Anders Fomsgaard; Tony Fooks; Ivo Foppa; Frode Forland; Anne Fouillet; Sandra Fournier; Greg Fox; Elisabetta Franco; Alexander Friedrich; Ingrid Friesema; Shouichi Fujimoto; Wolfgang Gaissmaier; Juan Carlos Galan; Gary Garber; Michel Garenne; Petra Gastmeier; Raphael Gaudin; Marius Gilbert; Rosina Girones; John Glasser; Shana Godfred-Cato; Pere Godoy; Emily Goldstein; Maureen Goss; Céline Gossner; Magnus Gottfredsson; Luigi Gradoni; Gilbert Greub; Margrethe Greve-Isdahl; Larisa Gubareva; Jan Gunst; Jean-Paul Guthmann; Bernardo Rafael Guzman Herrador; Katrine Bach Habersaat; Christos Hadjichristodoulou; Anette Hammerum; Sonja Hansen; Pia Hardelid; Sally Hargreaves; Heli Harvala; Joana Haussig; Craig Hedberg; Janneke Heijne; Joris Hemelaar; Rafael Herruzo; Markus Hilty; Hans Hirsch; Boris Hogema; Heidemarie Holzmann; Susan Hopkins; Rachel Hoyles; Wan-Ting Huang; Jim F Huggett; Anette Hulth; Hilary Humphreys; Daniel Hungerford; Michael Höhle; Judith Hübschen; Thomas Inns; Giuseppe Ippolito; Nazrul Islam; Esma Islamaj; Angela Iuliano; Alexandra Jablonka; Dorota Jamrozy; Claire Jenkins; Eric A Jensen; Silvia Jimenez-Jorge; Kari Johansen; Steven John; Helen Johnson; Terry Jones; Pernille Jorgensen; Laurence Josset; Charlotte S. Jørgensen; Sarah Kada; Kamran Kadkhoda; Alexander Kallen; Günter Kampf; Anu Kantele; Tommi Karki; Alyson Kelvin; Maria Keramarou; Liliane Keros; Marie E. Killerby; Pete Kinross; Esther Kissling; Don Klinkenberg; Gwen Knight; Anke Kohlenberg; Marion Koopmans; Uwe Koppe; Rolf Kramer; Florian Krammer; Tyra Krause; Gerard Krause; Annelies Kroneman; Adam Kucharski; Tanja Kuchenmuller; Peter Kuhnert; Ed J Kuijper; Michael Kurilla; Kin On Kwok; Kathryn E. Lafond; Filippo Lagi; Amparo Larrauri; Eric Lau; Jean-Philippe Lavigne; Jeffrey Lazarus; Quentin Le Hingrat; Jason J Leblanc; Jonggul Lee; Clara Lehmann; Sebastian Lequime; Tinne Lernout; Kathy Leung; Daniel Levy-Brühl; Bruno Lina; Theodore Liou; Marc Lipsitch; Yang Liu; Giovanni Lo Iacono; Anna-Leena Lohiniva; SW Long; Rohit Loomba; Nicola Low; Christian Lueck; Barbara M Lund; Åke Lundkvist; Xufei Luo; Theodore Lytras; Outi Lyytikäinen; Emma Löf; Renke Lühken; Pablo M De Salazar; Kristine Macartney; Ian Mackay; Gkikas Magiorkinis; Cecile Magis-Escurra; Benjamin Maier; Gannon C.K. Mak; Helena Maltezou; Michal Mandelboim; Annette Mankertz; Jean-Michel Mansuy; Josefa Masa-Calles; Mathieu Mateo; Sebastian Maurer-Stroh; Noel McCarthy; Gerald McInerney; Genevieve McKew; Lucy McNamara; Jolyon Medlock; Adam Meijer; Paula Meireles; Jacques Meis; Kassiani Mellou; Jeffrey Mercante; Lore Merdrignac; Mathias Merker; Stefano Merler; Silvia Meschi; Benjamin Meyer; Kai Michaelis; Jean Millet; Vivi Miriagou; Kenji Mizumoto; Chris Ka Pun Mok; Israel Molina; Jacob Moran-Gilad; Yamir Moreno; Clint Morgan; Keita Morikane; Shila Mortensen; Joël Mossong; Sandra Mounier-Jack; Vincent Munster; David Murdoch; K. Darwin Murrell; Michelle Murti; Mette Myrmel; Olaf Müller; Celine Nadon; Anna Nagy; Ndeindo Ndeikoundam Ngangro; Howard Needham; Joice Neves Reis Pedreira; Nathalie Nicolay; Jens Nielsen; Jessica Nihlén Fahlquist; Hiroshi Nishiura; Harold Noel; Paulo Nogueira; Hanna Nohynek; Torbjörn Norén; Angela Novais; Glen Nowak; Norbert Nowotny; Dennis Nurjadi; Dominik Sebastian Nörz; Anna Odone; Lulla Opatowski; W Paget; José Arthur Paiva; Takis Panagiotopoulos; Marcus Panning; Annalisa Pantosti; Anna Papa; John Papp; Gabriel Parra; Suzan Pas; Marie-Claire Paty; Lara Payne; Richard Pebody; Ana Penedos; Pasi Penttinen; Ranawaka A P M Perera; Ana Perez-Escoda; Unai Perez-Sautu; Martin Petric; Susanne Pfefferle; Yvonne Pfeifer; Anastasia Pharris; Gorben Pijlman; Johann Pitout; Diamantis Plachouras; Pedro Plans-Rubió; Evangelia D. Platsouka; Mateusz Plucinski; Catherine Plüss-Suard; Laurent Poirel; Chiara Poletto; Constantina Politis; Mario Poljak; Marina Pollan; Stefano Pongolini; Leo Poon; MahmoudReza Pourkarim; Andrew Preston; Natalie Prystajecky; Andrea Pugliese; Milo Puhan; Joan Puig-Barbera; Billy Quilty; Filip Raciborski; Barbara Rath; Andrainolo Ravalihasy; Raffaella Ravinetto; Vincenza Regine; Chantal Reusken; Ute Rexroth; Giovanni Rezza; Matteo Riccò; Daniel Richardson; Michael Rigby; Thomas V. Riley; Emmanuel Robesyn; Joacim Rocklöv; Angela Rose; Maiken Rosenstierne; Paul Rota; Adam Roth; Maja Rupnik; Katilyn Sadtler; Marc Saez; Linda Saif; Cristiano Salata; Gloria Sanchez; Sabine Santibanez; Gianpaolo Scalia-Tomba; Samuel Scarpino; Barbara Schimmer; Jonas Schmidt-Chanasit; Stefan Scholz; Madlen Schranz; Peter Schulz; Mitchell Schwaber; Sheree Schwartz; Ilan Schwartz; Ettore Severi; Anita Shah; Giri Shankar; Yehuda Shoenfeld; Aaron Siegler; Carlo Signorelli; Lotta Siira; Ian Simms; Andreas Sing; Anika Singanayagam; Mary Anissa Sinnathamby; Hans-Christian Slotved; Patrick Soentjens; Kristian Soltesz; Gianfranco Spiteri; Anneke Steens; Paola Stefanelli; James Stimson; Alexander J Stockdale; Heribert Stoiber; Maja Stosic; Julia Stowe; Sunita Sturup-Toft; Kanta Subbarao; Bertrand Sudre; Carl Suetens; Aya Sugiyama; Jonathan Suk; Sheena Sullivan; Patrick Sullivan; Arnfinn Sundsfjord; Piyarat Suntarattiwong; Makoto Takeda; Johanna Takkinen; Lara Tavoschi; Karin Tegmark-Wisell; Anders Tegnell; Peter Teunis; Robin Thompson; Craig Thompson; Andrew Thompson; Stacy Todd; Laura Toivonen; Kristin Tolksdorf; Sarah Tonkin-Crine; Karina Top; Nuria Torner; Arturo Torres Ortiz; Alma Tostmann; Ramona Trebbien; Tim Tsang; Sarah Tschudin-Sutter; Gro Tunheim; Soren Uldum; Helmut Uphoff; Palle Valentiner-Branth; Sophie Valkenburg; Birgit van Benthem; Gerrita van den Bunt; Kees. C. van den Wijngaard; Wim van der Hoek; Nicoline van der Maas; Kristien Van Reeth; Edward van Straten; Guido Vanham; Philippe Vanhems; A Vantarakis; Olli Vapalahti; Alkiviadis Vatopoulos; Aurelie Velay; Lasse Vestergaard; Cécile Viboud; Peter Vickerman; Russell M Viner; Leo Visser; Margreet Vos; Georgia Vourli; Bea Vuylsteke; Silja Vöneky; Benjamin Wachtler; Jacco Wallinga; Jan Walter; Gilles Wandeler; John Watkins; Scott Weese; Matthijs Welkers; Tobias Welte; Guido Werner; Therese Westrell; Andreas Widmer; Joanne Wildenbeest; Annelies Wilder-Smith; Emma Wiltshire; Christian Winter; Carl-Heinz Wirsing von König; Charles Wiysonge; Katja Wolthers; Anabelle Wong; Joseph Wu; Jianhong Wu; Roman Wölfel; Andrea Würz; Guogang Xu; Takuya Yamagishi; Jonathan Yoder; Dong Keon Yon; Barnaby Edward Young; Ioannis Zabetakis; Maria Zambon; Lorenzo Zammarchi; Philipp Zanger; Robert Zangerle; Peter Zarb; David Zella; Werner Zenz; Xu-Sheng Zhang; Walter Zingg; Phillip Zucs.

